# Influence of plasma-activated water treatment on the cold storage stability and flavor profile of duck fillets

**DOI:** 10.1016/j.fochx.2025.103321

**Published:** 2025-11-26

**Authors:** Shilong Ju, Junqi Li, Binghui Chen, Liyuan Niu, Daodong Pan, Yu Qian, Lihui Du

**Affiliations:** a Zhejiang Key Laboratory of Intelligent Food Logistic and Processing, State Key Laboratory for Managing Biotic and Chemical Threats to the Quality and Safety of Agro-products, College of Food Science and Engineering, Ningbo University, Ningbo 315211, China; b School of Civil and Environmental Engineering and Geography Science, Ningbo University, Ningbo 315211, China; c Zhejiang Jinwei Biotechnology Co., Ltd., Jiaxing 314299, China; d Key Laboratory of Cold Chain Food Processing and Safety Control, Zhengzhou University of Light Industry, Zhengzhou 450002, China

**Keywords:** Plasma-activated water, Duck fillets, Storage quality, Flavor profile

## Abstract

Plasma-activated water (PAW) is an emerging, nonthermal preservation technology with potential for meat products, though its application to duck fillets remains underexplored. This study investigated how PAW affects the storage quality and flavor of refrigerated duck fillets (4 °C). PAW treatment (0–6 min) significantly suppressed the total viable count (TVC), maintaining microbial load below the spoilage threshold for over 9 days. Mechanistically, the reactive oxygen and nitrogen species (RONS) in PAW simultaneously inhibit microbial proliferation and exert antioxidant activity. This dual action significantly suppressed both lipid oxidation and protein degradation, key processes of quality decay. Compared to controls, PAW enhanced water retention, reducing moisture loss by 67 %. Furthermore, PAW effectively preserved the flavor profile by inhibiting the formation of key off-odor compounds (e.g., 1-nonanol, butanal). These findings suggest PAW offers a clean-label, synergistic preservation strategy for duck fillets, positioning it as a highly competitive alternative to thermal techniques.

## Highlights

• *Staphylococcus saprophyticus* was the main spoilage bacteria in duck fillet.
• Plasma-activated water (PAW) inhibited spoilage of duck fillet during cold storage.
• PAW reduced the quality changes and lipid oxidation of duck fillet during storage.
• PAW inhibited the increase of 1-nonanol, butanal, and 1-octen-3-ol during storage.

## 1 Introduction

Duck meat is a highly nutritious poultry product, renowned for its rich composition of high-quality protein (18.6–20.8 %), abundant polyunsaturated fatty acids (accounting for 20–40 % of total fatty acids), and relatively low intramuscular fat content (ranging from 2.7 to 8.2 %) (da Silva Costa et al., 2023). As global awareness of health and nutrition continues to grow, duck meat has become an increasingly popular choice among consumers seeking nutritious and balanced meat options (Shin et al., 2023). Worldwide consumption of duck meat has grown rapidly, with a total production increasing from 4.4 million tons to 6.2 million tons in the past decade (year 2013–2021) (Islam et al., 2024; Liu & Churchil, 2022). This surge in duck meat production could potentially pose some challenges when not properly managed (Cordeiro et al., 2022; Shin et al., 2023). Microbial contamination during processing and storage leads to spoilage and foodborne illnesses, resulting in annual economic losses exceeding $90 billion (Betancourt Barszcz, 2024; Scharff, 2020). For instance, *Salmonella* infections are responsible for economic costs over $4.1 billion annually in the United States (Li et al., 2025).

In addition, the high moisture content of duck meat accelerates lipid oxidation and microbial proliferation, limiting its refrigerated shelf life to 3–5 days at 4 °C (Ungkusonmongkol & Wattanachant, 2024). Conventional preservation methods, such as thermal processing and chemical preservatives (e.g., sodium nitrite), are increasingly considered inadequate. Thermal treatments could degrade heat-sensitive nutrients (20–30 % loss of vitamin B12) and alter texture, while chemical additives face regulatory scrutiny due to carcinogenic nitrosamine formation risks (Deveci & Tek, 2024; Umair et al., 2022). Non-thermal alternatives including high-pressure processing (HPP), ultraviolet (UV) light and pulsed electric fields (PEF) have been gradually adopted in food processing to extend product shelf life. Nevertheless, there are some challenges that need to be overcome, such as high cost and flavor alterations (Martínez et al., 2023; Nath et al., 2023; Wang, Chen, et al., 2023). For example, disadvantages of HPP and PEF include long processing times, high equipment cost and unfavorable physical-chemical changes. Meanwhile, the nutritional loss and flavor deterioration caused by ultraviolet sterilization also raise concerns. Cold plasma (CP) is a non-thermal processing technology, which has the advantages of high antimicrobial efficiency and simple equipment. However, direct CP treatment is limited by certain drawbacks, including color loss, surface topography changes due to etching, and degradation of bioactive compounds (Zou et al., 2025). Therefore, further research is needed to exploit the properties of CP to expand its application in food production. Plasma-activated water (PAW), produced by exposing water to plasma discharge, contains reactive species including hydrogen peroxide, nitrite, and nitrate, and it has been utilized for microbial inactivation, seed germination, and protein modification as a non-direct CP treatment (Guragain et al., 2021; Hu et al., 2024; Wang, Zhou, et al., 2023). At present, the application of PAW in meat products research mainly focuses on its use in inactivating foodborne pathogens. For example, Sammanee et al. (2022) found that the microbial loads of *Campylobacter* spp. reduced 1.03 ± 0.99 log CFU/g in pork after PAW treatment. Kang et al. (2022) found that the population of *S. typhimurium* on chicken meats decreased by 1.23 lg CFU/cm^2^ after incubation in plasma-activated acetic acid for 10 min. Esua et al. (2020) observed that the microbial load of L. *monocytogenes* on grass carp decreased by 6.35 log CFU/g after plasma-functionalized buffer immersion. However, studies on plasma-activated water (PAW) for inactivating spoilage bacteria in duck meat are still lacking. Therefore, unlike previous studies limited to microbial inactivation, this work integrates microbiomics, lipid-protein oxidation, and volatile metabolomic analyses in a high-fat poultry system. By integrating advanced analytical techniques, including 16S rRNA sequencing, HS-SPME-GC–MS, and biochemical assays, we assessed the inhibitory effect of PAW on main spoilage bacteria on /duck fillets. The effects of PAW on stabilizing the integrity of lipids and proteins have been studied. Meanwhile, the ability of PAW to regulate volatile flavor compounds has been detected.

The objective of this research was to elucidate the dual antimicrobial and antioxidant roles of PAW in maintaining microbial and flavor stability of duck fillets during 4 °C storage. The dominant spoilage bacteria in duck fillet were identified in this study, and the PAW inactivation against dominant spoilage bacteria was evaluated at different meat storage times. Additionally, the effects of PAW on the quality attributes and key flavoring substances of duck fillets were analyzed. The findings of this study can provide the theoretical basis for the utilization of PAW, contributing to improved meat safety and quality.

## 2 Materials and methods

### 2.1 Reagents

Duck breasts were purchased from Henan Huaying Agricultural Development Co. Ltd. (Henan, China) and stored in the freezer at −20 °C before the experiment. Luria-Bertani (LB) broth and plate count agar (PCA) plates were brought from Hangzhou Microbial Reagent Company (Zhejiang, China). Protein Carbonyl assay kit (A087-1-2) was purchased from Nanjing Jiancheng Bioengineering Institute (Nanjing, China). Lowry Protein Assay Kit (PH0328) was acquired from Phygene Bio-Technology Co, Ltd. (Fujian, China). Meat seasonings were purchased from Jinchanba condiment shop (Henan, China). Other chemicals were all of analytical grade and obtained from Macklin (Shanghai Macklin Biochemical Co., Shanghai, China).

### 2.2 Identification of dominant spoilage bacteria from duck fillets

#### 2.2.1 Preparation of duck fillets and bacterial community structure analysis

Duck breast were cut into 5 × 1 × 0.5 cm fillets. The duck fillets (25 g) were accurately weighed and marinated for 24 h at 4 °C in a sterile sampling bag with tap water (50 mL) and meat seasonings (2 g). After marinating, Total genome DNA from duck fillets was extracted using CTAB/SDS method. DNA concentration and purity was monitored on 1 % agarose gels. According to the concentration, DNA was diluted to 1 ng/μL using sterile water. 16S rRNA genes of distinct regions were amplified used specific primer with the barcode. All PCR reactions were carried out with TransStart® FastPfu DNA Polymerase (TransGen Biotech). Mix same volume of 1× loading buffer (contained SYB green) with PCR products and operate electrophoresis on 2 % agarose gel for detection. PCR products was mixed in equidensity ratios. Then, mixture PCR products was purified with QIAquick@ Gel Extraction Kit (QIAGEN). Sequencing libraries were generated using SMRTbellTM Template Prep Kit (PacBio) following manufacturer's recommendations. The library quality was assessed on the Qubit@ 2.0 Fluorometer (Thermo Scientific) and FEMTO Pulse system. Finally, the library was sequenced on the PacBioSequel platform.

#### 2.2.2 Isolation of dominant spoilage bacteria

After marinating, 5.0 g of the duck fillets were homogenized in 45.0 mL of sterilized water using a flapping homogenizer. Then, 0.1 mL of the resulting mixtures were aseptically streaked onto PCA plates and incubated at 37 °C for 24 h. Ten single colonies were randomly extracted and serve as a template for PCR amplification. The PCR was carried out utilizing universal primers for the 16S rRNA gene 27F (5′-AGAGTTTGATCCTGGCTCAG-3′) and 1492 R (5′-GGTTACCTTGTTACGACTT-3′). The amplification was performed as follows: initial denaturation at 94 °C for 10 min; 32 cycles of 30 s at 94 °C, 30 s at 56 °C and 90 s at 72 °C; this was concluded with a final extension step at 72 °C for 5 min. PCR reaction contained 10 × buffer 2.5 μL, dNTP 2 μL, EasyTaq DNA polymerase 0.3 μL, each primer (10 μM) 0.5 μL, template DNA 1 μL, and ddH_2_O to the total volume of 25 μL. The amplicons were electrophoresed on 2.5 % agarose gels with a DNA band of approximately 1,500 bp in all isolates. Then the amplicons were sent to Hzykang company (Hangzhou, China) for sequencing. Dominant spoilage bacteria was identified by aligning sequences with the National Center for Biotechnology Information (NCBI) nucleotide database. Finally, the dominant spoilage bacteria *Staphylococcus saprophyticus* was purified and named *S·S* NBU722.

### 2.3 Effect of PAW on quality characteristics of duck fillet during storage

#### 2.3.1 Preparation of PAW and duck fillets

The PAW was prepared according to the method of Rao et al. (2023) with slight modifications. The device utilized in this experiment was designed by Nanjing Prospect Electronics Technology Co. (PSPT-JSPI-15, Nanjing, China). The device consists of a plasma generator, a rotating nozzle and an air compressor. The carrier gas was air, and the airflow and power were set to 28–32 L/min and 680–700 W, respectively. The plasma jet nozzle was accurately placed 4 cm above 50 mL of liquid (sterile water), and exposed to the plasma jet for 0 min, 2 min, 4 min,6 min (sterile water, PAW-2 min, PAW-4 min, PAW-6 min). Besides, acetic acid was used to adjust the pH of the sterile water to 3.1 as another control named acid water.

The duck fillets (25 g) were accurately weighed and added meat seasonings (2 g). Then duck fillets were marinated for 24 h at 4 °C in a sterile sampling bag with sterile water, acid water, PAW-2 min, PAW-4 min and PAW-6 min (50 mL), respectively.

#### 2.3.2 Optical emission spectroscopy (OES) of plasma discharge system

The active particle composition in the cold plasma was evaluated by the OES. The measurements were taken using the Black Comet C-25 spectrometer with an F600-UV–vis-SR fiber optic cable (StellarNet Inc., Tampa, Florida, USA) in the range of 190–850 nm. The spectrometer was kept 10 mm away from the rotating nozzle. The integration time was set to 500 ms. All the tests were repeated multiple times, and standard deviations were recorded to estimate the uncertainties of the respective tests.

#### 2.3.3 Strain cultivation and preparation of duck fillets

The *S·S* was cultured in LB broth at 37 °C for 12 h. After subculturing for two consecutive passages at 37 °C for 12 h, the cells were harvested and re-suspended in sterilized phosphate buffer saline (PBS). Optical density (OD) was adjusted to 0.15 ± 0.01. 5 mL suspension was sprayed onto the meat fillets. Then, the duck fillets (25 g) were marinated for 24 h at 4 °C in a sterile sampling bag with sterile water, acid water, PAW-2 min, PAW-4 min and PAW-6 min (50 mL) as described above and seasonings (2 g). After marinating, the duck fillets were stored at 4 °C for 0, 3, 6 and 9 days.

#### 2.3.4 Determination of pH and total viable count

The pH of the duck fillets were conducted according to the method described by Yuan et al. (2022), with minor modifications. To measure the pH of duck fillets, 5.0 g of the fillets were firstly homogenized in 45.0 g of sterilized water using a flapping homogenizer, and the resulting mixtures were filtrated to measure the pH of filtrate using a pH meter (PC5, Shanghai Sanxin Co., Ltd., China).

Viable counts of duck fillets were measured by plate counting method. After diluting the resulting mixtures according to a concentration gradient, 100 μL of each dilution was added to the LB agar. The plates were turned upside-down and kept at 37 °C for 24 h. Results were recorded as logarithms of the number of colony-forming units per g (log CFU/g).

#### 2.3.5 Determination of moisture content

To determine the moisture content, approximately 2 g of duck fillets were placed in a tray and the samples were measured a moisture meter (DHS-20 A, Shanghai Jingqi Co., Ltd., China).

#### 2.3.6 Determination of color

Color of duck fillets were detected according to the method of Zhu et al. (2022) with slight modifications. Color of duck fillets were detected using a colorimeter (SWG-2300, Shanghai Shuoguang Co., Ltd., China). Before the experiments, the colorimeter was calibrated with the calibration plate, and then the lightness (L*), redness (a*) and yellowness (b*) of the different duck fillets were recorded.

#### 2.3.7 Determination of malondialdehyde (MDA)

The MDA were identified as described by Tang et al. (2025) with minor adjustments. The duck fillets (5 *g*) were placed in centrifuge tubes, followed by the addition of 45 mL of trichloroacetic acid and homogenized at 10,000 rpm for 30 s using a homogenizer. After centrifugation at 4000*g* for 15 min, 2.5 mL of the supernatant was mixed with 2-Thiobarbituric acid (2.5 mL, 0.02 mol/L). The mixture was incubated in 90 °C for 30 min. Afterward, the mixture was cooled in water for 10 min and the absorbance of the supernatant at 532 nm was measured using a spectrophotometer.

#### 2.3.8 Determination of total volatile basic nitrogen (TVB-N)

The TVB-N of samples were determined using a automatic Kjeldahl nitrogen analyzer following the method with slight modifications Cao et al. (2024). The duck fillets samples (5 g) were dispersed in 45 mL of deionized water and homogenized at 10,000 rpm for 30 s using a homogenizer. The boric acid (10 mL) and mixed indicator liquid (5 mL) were added to the distillation condensing tube and distilled for 5 min before titration with HCl (0.10 mol/L) until the color changed to blue-purple.

#### 2.3.9 Determination of total sulfhydryl contents and carbonyl contents

##### 2.3.9.1 Extraction of MPs

The myofibrillar proteins were extracted using a slightly modified version of the described method Li et al. (2024). The duck fillets (5 g) were suspended in 4 volumes (*w*/*v*) of extraction buffer (100 mM Tris, 10 mM EDTA, pH 8.3) and centrifuged at 6000×*g* for 20 min. The precipitates were re-suspended in standard salt solution (100 mM KCl, 20 mM Na_2_HPO_4_/NaH_2_PO_4_, 2 mM MgCl_2_, 1 mM EGTA, pH 7.0) at 4 volumes (w/v). The precipitates were suspended in extraction buffer three times. After the third suspension, the precipitates were washed twice with standard salt solution containing 1 % Triton X-100. Then the precipitates were dissolved in 100 mM KCl and filtered through coarse cotton cloth. Each step included centrifugation at 8000×*g* for 10 min. The precipitates were dissolved in 100 mM NaCl, centrifuged, and washed with deionized water. The final MP precipitates were stored at 4 °C and the MP concentration was adjusted to 5 mg/mL using the Biuret method.

##### 2.3.9.2 Total sulfhydryl contents assay

Total sulfhydryl contents were analyzed following Cheng et al. (2022) with slight modifications.0.5 mL MPs solution (5 mg/mL) was taken and 2.5 mL Tris-HCl buffer (0.086 M Tris, 0.09 M glycine, and 4 mM EDTA, pH 8) was added. 3 mL of the mixture was mixed with 0.02 mL of 0.1 % 5,5′ -dithio-bis (2-nitrobenzoic acid) (DTNB) and incubated at 25 °C for 30 min. DTNB solution was used as blank, and the absorbance at 412 nm was measured using an ultraviolet visible spectrophotometer, and the total sulfhydryl contents were calculated as follows:

$$\text{Total sulfhydryl contents}=73.53\times \left(A-A_{0}\right)\times D/C$$

“A” represents the absorbance of the samples at 412 nm, “A_0_” refers to the absorbance of the 0.6 M KCl solution at 412 nm, and C represents dilution ratio of samples.

##### 2.3.9.3 Carbonyl groups assay

The carbonyl contents were measured using Protein Carbonyl assay kit according to the manufacturer's instructions.

### 2.4 Identification and analysis of volatile compounds

The volatile compounds in duck were detected using HS-SPME-GC–MS (8890 GC System+5977B/MSD, Agilent, USA) according to the method of Y. Cheng et al. (2024), with slight modifications. 5 g of duck fillets were placed in a headspace extraction vial and extracted using a 75 μm Carboxen/poly dimethylsiloxane solid phase microextraction (SPME) fiber at 55 °C for 60 min, then the SPME fiber was injected into the GC injection port at 250 °C, and the volatiles were separated on a VFCOL capillary column (30 m × 250 μm × 0.25 μm). The initial temperature of 35 °C was maintained for 3 min, the increased to 140 °C at 4 °C/min for 1 min, and finally to 250 °C at 10 °C/min for 3 min. Helium was the carrier gas at 1 mL/min. Mass spectra were generated in electron impact (EI) mode (0.9 kV) with a scanning mode of *m*/*z* 45–600. The NIST14 data base was used to identified the compounds by comparing their retention times and mass spectra.

### 2.5 Statistical analysis

All tests were performed in three independent biological replicates (different duck fillets). The results of the test were presented as the mean ± standard deviation (SD). Statistical analyses were performed using a one-way analysis of variance (SPSS 22.0). Significance was defined as (*P < 0.05*).

## 3 Results and discussion

### 3.1 Isolation of dominant spoilage bacteria

**Fig. 1:**
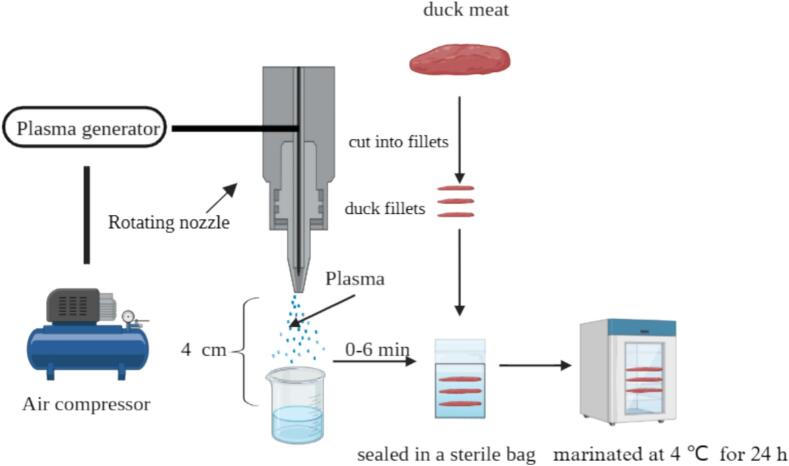
Diagram of PAW treatment experimental setup and conditions for curing duck meat.

**Fig. 2:**
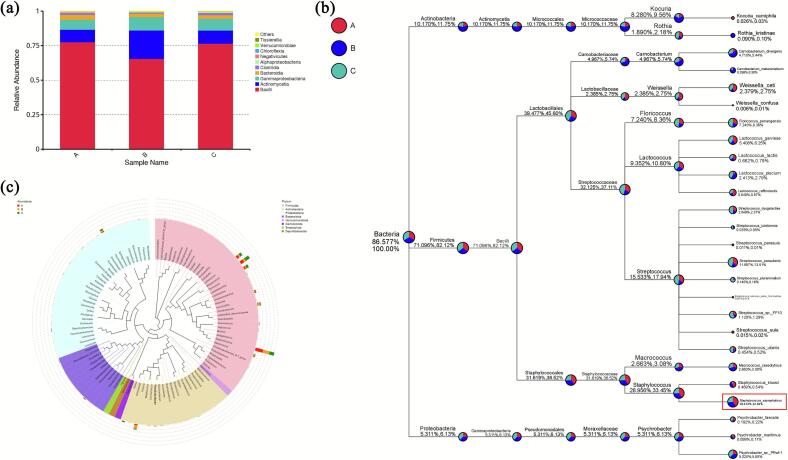
(a): Relative abundance of species at phylum level of duck fillets after marinating. Letters A, B, and C represented the parallel samples taken at the same time point (after 24 h of marination at 4 °C). (b): Taxonomic tree for specific species of duck fillets after marinating. (c): Genus level species evolutionary tree of duck fillets after marinating.

To identify the dominant spoilage strain in duck fillets, the 16S rRNA gene amplicon sequencing was employed to analyze the composition of the microbial community. As presented in Table 1, the coverage of all groups exceeded 0.99, indicating that the sequencing results accurately reflected the microbial profile of the duck fillets. Operational Taxonomic Units (OTUs), which are classification units acquired by clustering sequences based on similarity (above 97 %), were used to assess species richness and diversity within the community (Chen et al., 2025). The Chao index is an index that estimates the number of OTUs in a community using the chao1 algorithm, and it has been widely used in ecology to estimate the total number of species. Bacterial diversity was evaluated using α-diversity indices, including the Chao, Simpson, and Shannon indices, with higher values for these indices indicate greater community diversity (Hou et al., 2023). At the phylum level, *Firmicutes*, *Actinobacteria*, and *Proteobacteria* were the most abundant, with *Firmicutes* being the predominant phylum (Fig. 2a). Similar results have been observed in previous researches (Lyu et al., 2021; Zhou et al., 2022). As illustrated in Fig. 2b, the analysis of the microbial community within the duck fillets revealed a composition overwhelmingly dominated by Bacteria, which accounted for 86.58 % of the classified sequences. Among these, the phylum *Firmicutes* was predominant, representing 71.10 % of the total microbial abundance. Major taxa included members of the orders *Staphylococcales* and *Lactobacillales*, with the family *Staphylococcaceae*—particularly the genus *Staphylococcus* (28.96 %)—being the most abundant. *Staphylococcus saprophyticus* emerged as the most abundant bacterial species, accounting for 28.43 % of the total microbial population. These results conclusively demonstrated that *Staphylococcus saprophyticus* was the dominant spoilage bacterium in duck fillets. (See Fig. 1.)

**Table 1:** Alpha Indices table.

Group	OTUs	Shannon	Simpson	Chao1	Coverage
A	226	4.27	0.88	266.14	0.997
B	229	4.36	0.90	231.88	0.999
C	292	4.22	0.85	320.56	0.999

### 3.2 Optical emission spectroscopy (OES)

**Fig. 3:**
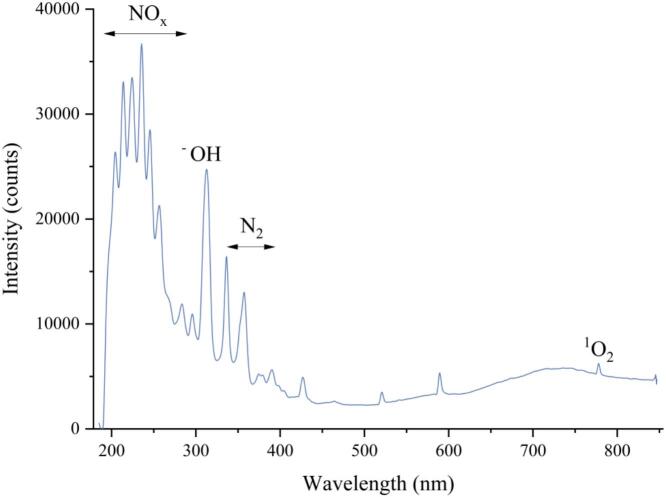
Optical emission spectra peaks obtained during CP treatment.

OES was used to identify the active particles responsible for CP. As shown in Fig. 3, the emission spectrum of CP was detected within the 190–850 nm range. The characteristic peak (200–280 nm) is related to the formation of NO and NO_2_. Meanwhile, the peaks of ·OH and singlet state oxygen were observed at 308.5 nm and 778 nm. Those peaks suggest that the active species of CP utilized in this study mainly comprised of ROS (reactive oxygen species) and RNS (reactive nitrogen species). Similar findings were also reported in the study conducted by Liu et al. (2024), where active species consisting of ·OH and singlet oxygen were detected within the above wavelength range. Our previous work measured the concentration of the active substances under the same treatment conditions. The results showed that the main active substances (hydrogen peroxide, nitrite and nitrate) in PAW were up to 108.00 μmol/L, 1300.23 μmol/L and 2396.04 μmol/L at 6 min treatment, respectively.

### 3.3 Changes in total viable count (TVC) and pH of the duck fillets during storage

**Fig. 4:**
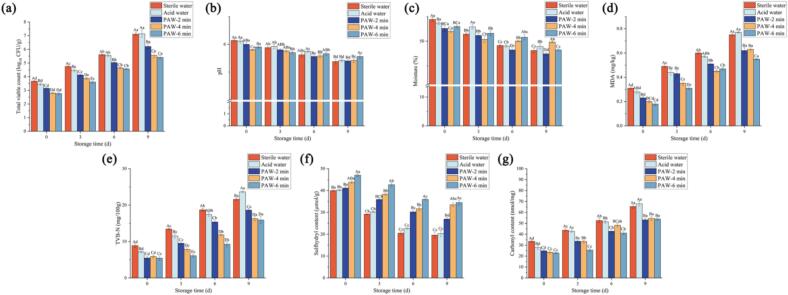
Quality changes of duck fillets during storage at 4 °C: (a) Changes in the viable count of *S. S* during storage. (b): Changes in the pH values of duck fillets during storage. (c): Changes in the moisture of duck fillets during storage. (d): Changes in the MDA contents of duck fillets during storage. (e): Change of TVB-N of duck fillets during storage. (f): Changes in the sulfhydryl contents of duck fillets during storage. (g): Changes in the carbonyl content of duck fillets during storage.

Microbial proliferation is a critical factor in assessing the quality and shelf life of food products (Zhang et al., 2022). TVC changes in the duck fillets during storage are displayed in Fig. 4a. The results revealed a significant (*p* < 0.05) increase in TVC across all groups over the storage period. This trend can be attributed to the utilization of nutrients, such as free amino acids and water-soluble proteins, by microorganisms for growth and reproduction, which is consistent with the findings reported by Z. Song et al. (2023). The initial TVC of all PAW-treated groups ranged from 2.86 to 3.15 log CFU/g, whereas the sterile water and acid water groups exhibited higher initial TVC of 3.67 log CFU/g and 3.45 log CFU/g, respectively. Notably, the PAW-treated groups demonstrated a slower increase in TVC with the extension of PAW generation time. Compared to acidic water, PAW demonstrates a stronger antibacterial ability. PAW may originate from the oxidative ability of the active substances. This can be explained by the ability of PAW to disrupt bacterial cell membranes, leading to the leakage of intracellular contents and subsequent bacteriostatic effects (Huang, Sun, et al., 2024). At day 9, the TVC in the PAW-6 min and PAW-4 min groups remained below 6.00 log CFU/g, which was in accordance with the Food Safety and Inspection Service Stabilization Guidelines for Meat and Poultry Products for the measurement of total colony counts in ready-to-eat meat. In contrast, the sterile water and acid water groups reached TVC of 7.12 log CFU/g and 7.14 log CFU/g, respectively. These findings highlighted the inhibitory effect of PAW on the growth of *S·S* NBU722 in duck fillets, effectively delaying spoilage and extending the shelf life of the product.

The pH value plays a crucial role in meat product, exerting a significant impact on color, water retention, and shelf life (Andrés-Bello et al., 2013). The effects of PAW treatment on the pH of duck fillets are presented in Fig. 4b. A significant decrease (*P < 0.05*) in pH was observed in duck fillets treated with PAW (PAW-6 min and PAW-4 min) compared to the control groups (sterile water and acid water groups). The reduction in pH can be attributed to the reaction between reactive species generated by CP treatment and the liquid matrix, leading to the formation of acidic compounds (Jiang et al., 2024). Gebremical et al. (2023) suggested that the low pH of PAW is associated not only with the generation of acidic H_3_O^+^ from water molecules and hydrogen peroxide but also with the formation of nitrite acid (HNO_2_) and nitric acid (HNO_3_) from nitrogen substances. During storage, the pH values in all treatment groups gradually decreased, consistent with findings reported by M. Song et al. (2024). This decrease in pH might result from the progressive oxidation of fats, resulting in the formation of free fatty acids, as well as the deamination of amino acids. Additionally, organic acids produced by microbial metabolism further contributed to the decline in pH. Notably, the pH reduction was slower in PAW-treated samples, which may be linked to the lower TVC observed in these groups, thereby highlighting the antimicrobial efficacy of PAW.

### 3.4 Changes in moisture content of the duck fillets during storage

The water content is another key quality parameter of meat, as it is related to sensory and textural characteristics the microbial growth and storage stability. It is well established that maintaining optimal water content in meat is essential for inhibiting the growth of microorganisms, including fungi and mold Feng (2022); Mediani et al. (2022). As depicted in Fig. 4c, PAW treatment significantly reduced the moisture content (*P < 0.05*), with the lowest moisture content observed in the PAW-4 min group at day 0. This reduction can be attributed to the structural alterations in the meat induced by PAW treatment Du et al. (2025). The decline in moisture content primarily resulted from the disruption of muscle structures caused by PAW. Over the storage period, a slight decrease in moisture content was observed across all groups, which is due to the oxidative degradation of myofibrillar proteins, which further compromised muscle integrity Tao et al. (2021). The moisture contents in sterile water group decreased by 6.3 % in 9 days, while that of the PAW-4 min group decreased by only 2.17 %. The reactive species (reactive oxygen and nitrogen species) in PAW may induce cross-linking of proteins, forming a denser matrix that limited further water migration. Additionally, nitrites in PAW also stabilize myoglobin and chelate pro-oxidative metal ions (Zang et al., 2023). These structural modifications preserve the water-holding capacity of the meat and enhancing its storage stability.

### 3.5 Changes in color of the duck fillets during storage

Color is a critical quality attribute of food, significantly influencing consumer preference and purchasing decisions (Altmann et al., 2022). To evaluate color changes in duck fillets during storage, colorimetric parameters including L* (lightness), a* (redness/greenness), and b* (yellowness/blueness) were measured. Table 2 reveals the visual color changes in duck fillets over storage period. Compared to the control groups, the L* values of PAW-treated duck fillets exhibited a significant decrease (*P* < 0.05). The decline in L* values may be caused by the decrease of moisture content and myoglobin oxidation. While the b* values were no significant differences among all the groups, the a* values were significantly higher in PAW-treated duck fillets (*P < 0.05*). The increase in a* values may be attributed to the denaturation of the globin moiety of myoglobin (Palanisamy et al., 2024). Ke et al. (2024) reported that nitric oxide (NO) in PAW can combine with myoglobin to form nitrosomyoglobin, a stable pink complex, which may explain the observed increase in a* values following PAW treatment. Meanwhile, the interaction effect of storage time on a* values was not significant, demonstrating that PAW treatment effectively preserved the redness of duck fillets throughout the 9-day storage period. The L* values gradually declined during storage, which may be attributed to heme oxidation in the meat and the influence of oxygen content, leading to chemical reactions in myoglobin and brown off-colors, which was consistent with previous research (Pogorzelska et al., 2018). In contrast, no significant changes were observed in the b* values across all samples over the storage period. These findings highlight the potential of PAW treatment to maintain the desirable color attributes of duck fillets during storage.

**Table 2:** Effect of PAW on the color of duck fillets during cold storage.

	Storage time (d)	Treatment conditions				
		Sterile water	Acid water	PAW-2 min	PAW-4 min	PAW-6 min
L*	0	63.66 ± 1.01^Aa^	61.59 ± 1.23^Ba^	57.97 ± 0.59^Da^	59.48 ± 0.52^Ca^	55.91 ± 0.70^Ea^
	3	60.17 ± 0.38^Ab^	58.72 ± 0.29^Bb^	56.99 ± 0.59^BCb^	55.66 ± 0.12^Cb^	55.04 ± 1.11^Cab^
	6	59.91 ± 0.44^Ab^	55.33 ± 0.54^BCc^	55.97 ± 1.43^Bb^	55.94 ± 1.38^BCb^	54.19 ± 0.55^Cb^
	9	56.05 ± 0.25^Bc^	55.07 ± 0.58^Cc^	57.90 ± 0.77^Aa^	52.12 ± 0.63^Dc^	55.15 ± 0.41^Cab^
a*	0	5.73 ± 0.33^Da^	5.94 ± 0.78^Db^	10.21 ± 0.55^Cb^	13.18 ± 0.48^Aa^	12.67 ± 0.18^Bb^
	3	4.85 ± 0.56^Db^	6.74 ± 0.86^Ca^	11.82 ± 0.51^Ba^	11.92 ± 0.33^Bb^	14.68 ± 0.67^Aa^
	6	4.87 ± 1.22^Cb^	5.76 ± 0.10^Cb^	11.73 ± 0.76^Ba^	13.28 ± 1.03^BCa^	14.23 ± 1.09^Aa^
	9	4.06 ± 0.42^Cc^	3.95 ± 0.65^Cc^	9.68 ± 0.25^Bc^	10.45 ± 0.56^ABc^	11.27 ± 1.02^Ac^
b*	0	19.00 ± 0.34^Aa^	17.18 ± 1.32^Ca^	18.57 ± 0.50^Ba^	18.80 ± 1.09^Aa^	16.14 ± 0.64^Ca^
	3	18.74 ± 0.61^Aa^	16.94 ± 0.67^BCb^	17.50 ± 0.26^Bb^	17.17 ± 1.45^Bb^	15.18 ± 0.86^Cb^
	6	17.58 ± 1.21^Bb^	17.19 ± 0.31^Ba^	18.59 ± 1.33^Aa^	17.43 ± 0.37^Bb^	15.10 ± 0.21^Cc^
	9	15.44 ± 1.42^Cc^	14.73 ± 1.23^Cc^	17.32 ± 0.98^ABb^	18.69 ± 0.54^Aa^	16.22 ± 1.01^Ba^

Note: L*: lightness; a*: redness; b*: yellowness; ^A-E^ indicates within the same row with different uppercase letters differ significantly (*P < 0.05*) (differences among the treatment conditions). ^a-d^ indicates within the same column with different lowercase letters differ significantly (*P < 0.05*) (differences between the storage time).

### 3.6 Changes in malondialdehyde of the duck fillets during storage

MDA, a byproduct of polyunsaturated fatty acid degradation, is associated with the development of off-odors and rancid off-flavors in meat (Pogorzelska et al., 2018). The extent of lipid oxidation and meat freshness can be assessed by measuring MDA levels using the TBARS assay, a key biomarker for lipid oxidation (Ponnampalam et al., 2017). Significant differences in MDA values were observed among all treated samples at storage times of 0, 3, 6, and 9 days (Fig. 4d). For example, at day 0, the TBARS values for the sterile water and PAW-6 min groups were 0.31 and 0.18 mg/kg, respectively, indicating that PAW treatment effectively reduced MDA content. This finding contrasts with the results of Qian et al. (2021), who reported that PAW accelerated lipid oxidation in chicken meat due to the generation of peroxides. However, as noted by Wu et al. (2022), the rate of lipid oxidation is influenced by the food matrix composition and the specific conditions of PAW treatment. In this study, PAW treatment demonstrated a protective effect against lipid oxidation in duck fillets, which is consistent with findings from other PAW-related studies (Chaijan et al., 2022; Xiao et al., 2023). This antioxidant effect may be attributed to the presence of nitrites in PAW, which bind to the iron center of myoglobin, reducing the availability of free iron and thereby inhibiting lipid oxidation (Premi et al., 2024). These results emphasize the potential of PAW as a treatment to preserve the lipid stability and freshness of duck fillets during storage.

### 3.7 Changes in TVB-N of the duck fillets during storage

TVB-N is widely recognized as a key indicator of muscle tissue deterioration, with its increase closely linked to the activity of spoilage bacteria and endogenous enzymes (Zhao et al., 2018). As shown in Fig. 4e, the initial TVB-N values indicated that the duck fillets were of high quality, consistent with the relatively low initial TVC. During storage, the TVB-N values of all samples increased significantly (*p < 0.05*). At day 9, the PAW-6 min and PAW-4 min samples exhibited significantly lower TVB-N values of 15.91 mg/100 g and 16.3 mg/100 g, respectively, compared to the sterile water and acid water groups, which reached higher levels of 21.59 mg/100 g and 23.65 mg/100 g (*p < 0.05*). The slower increase in TVB-N values in the PAW-treated samples may be attributed to the bacterial damage induced by PAW, which delayed microbial proliferation (Gao et al., 2024). These results suggest that PAW effectively inhibits TVB-N production, likely due to its inherent antimicrobial properties (Sun et al., 2023). Similar results have been reported in studies investigating the effects of PAW on TVB-N values in various food products (Chaijan et al., 2021; Liao et al., 2018).For instance, W. Zhu et al. (2023) observed that PAW treatment reduced the TVB-N values of salmon fillets from 17.27 mg/100 g to 12.00 mg/100 g following PAW treatment. These findings further support the efficacy of PAW in preserving the quality of meat products by mitigating spoilage-related biochemical changes.

### 3.8 Changes in sulfhydryl groups and carbonyl groups of the duck fillets during storage

Sulfhydryl (SH) groups and carbonyl groups were analyzed to assess changes in protein oxidation in duck fillets. Protein oxidation in meat is characterized by an increase in carbonyl groups and a decrease in SH groups (Bao & Ertbjerg, 2019; Fu et al., 2019). The reduction in SH content is primarily attributed to the formation of sulfur oxidation products and oxidation of disulfide bonds (Mishanina et al., 2015). Among all treatment groups, the PAW-6 min group exhibited the highest SH content at 47.04 μmol/g sample, followed by the PAW-4 min group. This observation indicated that PAW treatment effectively mitigated protein loss during storage. PAW treatment facilitated the exposure of hydrophobic protein groups and enhanced protein-protein interactions, thereby reducing the rate of protein oxidation (Qian et al., 2022). These findings are consistent with previous studies, which suggest that PAW maintains protein stability and delays protein oxidation **(**Huang, Liao, et al., 2024). Conversely, carbonyl group content decreased following PAW treatment. Carbonyl groups are generated through protein oxidation, leading to alterations in protein conformation **(**Zavadskiy et al., 2022). Specific amino acids such as threonine, proline, and lysine are oxidized to produce γ-glutamic semialdehyde and α-amino adipic semialdehyde, which are key carbonyl compounds associated with protein oxidation (Zavadskiy et al., 2022). PAW treatment reduced the exposure of protein functional groups, thereby inhibiting the formation of carbonyl groups (Tan et al., 2024). The results reveal the potential of PAW to preserve protein integrity and minimize oxidative damage in duck fillets during storage.

### 3.9 Effects of PAW on volatile flavor profiles in duck fillets

**Fig. 5:**
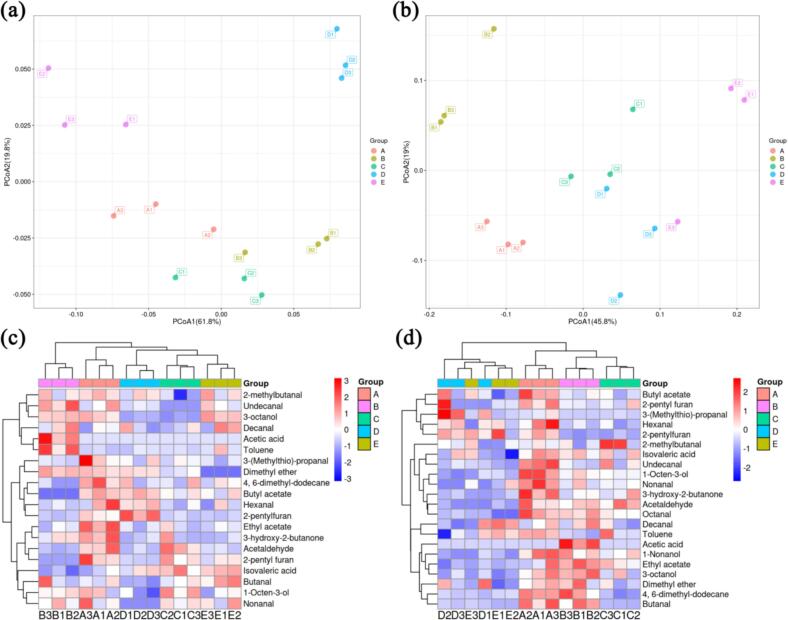
Letters A, B, C, D and E represented the different duck fillets groupings, they were respectively represented duck fillets treated with sterile water, acid water, PAW-2 min, PAW-4 min, PAW-6 min. These groups were analyzed at both day 0 and day 9 of storage. (a): PCA score plot of volatile substances at day 0. (b): PCA score plot of volatile substances at day 9. (c): The heat map of volatile metabolites at day 0. (d): The heat map of volatile metabolites at day 9.

Gas chromatography-mass spectrometry (GC–MS) was employed to analyze the volatile flavor components in duck fillets. A total of 23 flavor compounds were identified, including aldehydes, alcohols, esters, ketones, acids, and alkenes (Table 3). Principal Component Analysis (PCA) was utilized to visualize metabolic differences among the five groups of duck fillets. At day 0, the duck fillets from groups E and D (treated with PAW-4 min and PAW-6 min, respectively) were distinctly separated from the other three groups, indicating notable differences in metabolite profiles (Fig. 5a). The significant differences in metabolites, which were likely attributable to the presence of nitrite in PAW, as nitrite contributed to preservation and delayed the formation of undesirable odors. Previous studies have reported that nitrite is a widely used curing agent and plays a crucial role in preservation (Honikel, 2008; Parthasarathy & Bryan, 2012). Similar clustering patterns were observed at day 9, further highlighting the significant metabolic differences induced by PAW treatment. Groups A and B, treated with sterile water and acid water, respectively, were clearly separated from the other groups. This separation may be attributed to the high levels of corruption observed in these groups, as indicated by their higher TVB-N and TBARS values, which suggested a greater degree of deterioration compared to the PAW-treated groups.

**Table 3:** The compositional table of identified volatiles with retention times, compound class, and odor descriptors.

Name of substance	Retention time	Odor	CAS
**Aldehydes**			
Acetaldehyde	3.67	Fruity	75-07-0
Hexanal	3.58	Grass, tallow	66-25-1
Nonanal	11.83	Citrus, green	124-19-6
Decanal	13.17	Soap, orange	112-31-2
2-methylbutanal	6.24	Malt	96-17-3
Butanal	5.98	Pungent, green	123-72-8
Undecanal	10.51	Oil, sweet	112-44-7
3-(Methylthio)-propanal	5.63	Intense, onion	3268-49-3
Octanal	8.91	Soap, lemon	124-13-0
**Alcohol**			
1-Nonanol	14.12	Floral, green	143-08-8
1-Octen-3-ol	7.66	Mushroom	3391-86-4
1-Pentanol	8.92	Fusel, alcoholic	71-41-0
3-octanol	9.84	Moss, nut	20296-29-1
**Esters**			
Ethyl acetate	3.74	Pineapple	141-78-6
Butyl acetate	4.21	Pear	123-86-4
**Acids**			
Acetic acid	2.48	Sour	64-19-7
Isovaleric acid	5.25	Acid, rancid	503-74-2
**Others**			
Dimethyl ether	3.09	Etheric odor	115-10-6
3-hydroxy-2-butanone	6.87	Sweet, creamy	513-86-0
4,6-dimethyl-dodecane	12.41	Alkane	61141-72-8
2-pentyl furan	7.83	Bean, butter	3777-69-3
Toluene	8.67	Pungent	108-88-3

The heatmap further elucidated the relative abundance of differential metabolites across groups (Fig. 5c). Metabolites such as ethyl acetate, 3-hydroxy-2-butanone, and acetaldehyde were exclusively detected in group A. These compounds have been previously identified as contributors to undesirable odors (Bleicher et al., 2022; Wan et al., 2023; Wang et al., 2022). By day 9, duck fillets in the sterile water and acid water groups exhibited higher levels of spoilage, as evidenced by a significant increase in the concentrations of compounds such as 1-nonanol, Butanal, and 1-octen-3-ol. These substances have been recognized as biomarkers of spoilage in various studies (Mikrou et al., 2021; Wang, Wang, et al., 2025; Zhao et al., 2024). In contrast, the levels of butanal and 2-pentylfuran were observed in the PAW-treated groups. Previous research has indicated that these compounds are generated through lipid oxidation and protein degradation during storage (Shahidi & Oh, 2020; Wang et al., 2024; Wang, Zhou, et al., 2025). Based on these findings, we hypothesized that PAW treatment does not effectively delay the formation of furans and aldehydes, suggesting that these oxidative processes may still occur despite the antimicrobial effects of PAW.

**Fig. 6:**
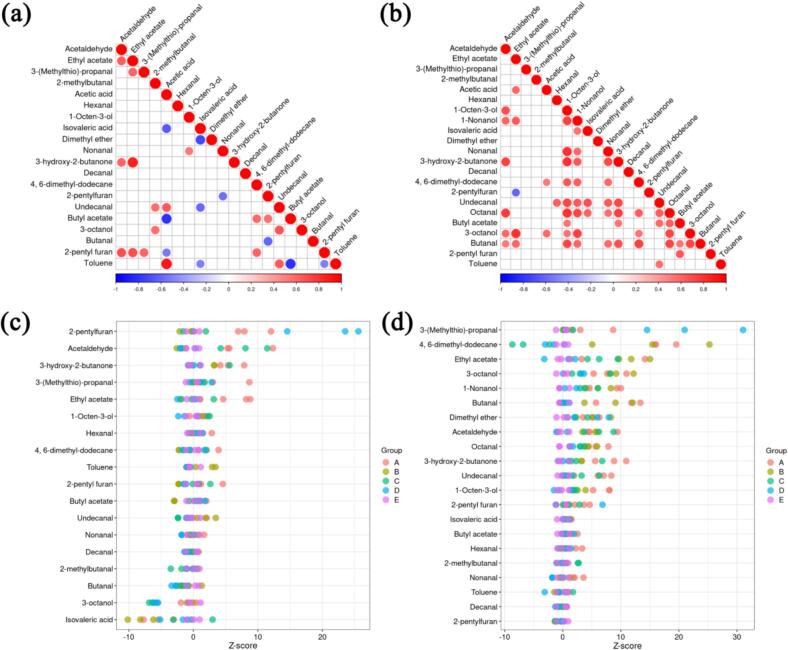
(a): Correlation matrix of metabolites across the groups at day 0. (b): Correlation matrix of metabolites across the groups at day 9. (c): *Z*-scores of metabolites across the groups at day 0. (d): Z-scores of metabolites across the groups at day 9.

**Fig. 7:**
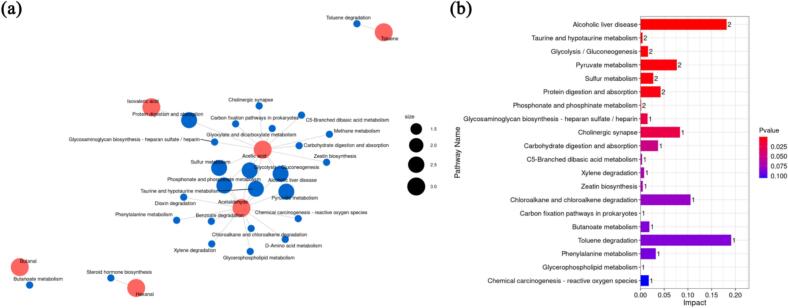
(a): Hierarchical cluster analysis of the enrichment level of KEGG pathway. (b): Metabolic pathway enrichment analysis.

Correlation analysis of metabolites (Fig. 6a and b) revealed how PAW modulates the volatile flavor network. For instance, 3-hydroxy-2-butanone and ethyl acetate exhibited a positive correlation at day 0, indicating that an increase in one compound was associated with a corresponding increase in the other. By day 9, the correlation among various metabolites became more pronounced due to the spoilage observed in the samples. Aldehydes such as octanal and undecanal, which are derived from lipid oxidation, showed a strong correlation, reflecting their shared origin from oxidative degradation of fats. Similarly, alcohols like 1-nonanol and 1-octen-3-ol, along with octanal, are produced through the further reaction of aldehydes with acids, as evidenced by their strong correlation with acetaldehyde. These findings proved the interconnected pathways of flavor compound formation and the influence of PAW on these metabolic processes. (See Fig. 7.)

The *Z*-score, a statistical measure standardized based on the relative quantitative values of metabolites, enables cross-comparison of metabolite levels across samples **(**Schober et al., 2023). At day 0, compounds including 2-pentylfuran, isovaleric acid, and acetaldehyde were enriched across all groups. However, by the end of storage, 3-(methylthio)-propanal and 4,6-dimethyldodecane emerged as dominant substances, reflecting significant shifts in the relative abundance of key metabolites during storage. Notably, Z-scores of metabolites in PAW-treated groups remained lower than those in the two control groups. For instance, 3-(methylthio)-propanal, a compound primarily derived from lipid oxidation **(**Luo et al., 2024), exhibited reduced prominence in PAW-treated samples compared to controls, suggesting attenuated oxidative processes in these groups. Based on the aforementioned observations, the TBARS values increased with the storage time, indicating that fats were continuously oxidized. In terms of flavor, the low concentration of aldehydes produced by fat oxidation reflected that PAW treatment could retained the flavor of duck fillets.

Kyoto Encyclopedia of Genes and Genomes (KEGG) enrichment analysis was performed to investigate the biochemical metabolic and signal transduction pathways associated with the identified metabolites. Compounds such as butanal, hexanal, and isovaleric acid were linked to multiple potential pathways, suggesting their diverse roles in spoilage processes. Additionally, toluene degradation emerged as a significantly influenced pathway. Toluene, which may originate from poultry feed, is likely metabolized by ducks and retained in their meat. While previous studies have reported that PAW can degrade benzene-related compounds **(**Shang et al., 2021), this study did not observe similar effects, indicating potential differences in metabolic pathways or PAW interaction mechanisms under these experimental conditions.

## 4 Conclusion

Our study established *Staphylococcus saprophyticus* as the predominant spoilage microorganism in duck fillets. PAW exhibited remarkable antibacterial activity, effectively reducing the total viable count of *S. S* during 4 °C storage. Furthermore, PAW effectively reduced lipid and protein oxidation, and mitigated flavor deterioration, thereby extending duck fillet shelf life during refrigeration. Collectively, these integrated findings highlight PAW as an innovative non-thermal intervention for simultaneously extending shelf life and maintaining organoleptic quality in refrigerated poultry products. However, the physical and chemical differences of various meat products may affect the applicability of PAW. Meanwhile, the understanding of reactions between PAW and bacteria at the molecular level should be developed. Our future research will systematically explore the adaptability of PAW processing parameters to different meat products and analyze its bactericidal mechanism.

## CRediT authorship contribution statement

**Shilong Ju:** Writing – original draft, Methodology, Investigation, Formal analysis. **Junqi Li:** Writing – review & editing, Data curation. **Binghui Chen:** Supervision, Investigation. **Liyuan Niu:** Writing – review & editing, Conceptualization. **Daodong Pan:** Resources, Conceptualization. **Yu Qian:** Writing – review & editing, Supervision. **Lihui Du:** Writing – review & editing, Validation, Supervision, Project administration, Funding acquisition, Conceptualization.

## Declaration of competing interest

The authors declare that they have no known competing financial interests or personal relationships that could have appeared to influence the work reported in this paper.

## Data Availability

Data will be made available on request.
